# Unraveling genetic diversity and population structure of pineapple germplasm using genome-wide SNP markers

**DOI:** 10.1007/s00438-025-02275-1

**Published:** 2025-07-19

**Authors:** Haomin Lyu, Tracie Matsumoto, Qingyi Yu

**Affiliations:** 1 Tropical Plant Genetic Resources and Disease Research Unit, Agricultural Research Service, U. S. Department of Agriculture, Daniel K Inouye U.S. Pacific Basin Agricultural Research Center, 64 Nowelo Street, Hilo, HI 96720 USA; 2 Hawaii Agriculture Research Center, Kunia, HI 96759 USA

**Keywords:** Pineapple, *Ananas comosus*, SNP, Heterozygosity, Population structure

## Abstract

Pineapple (*Ananas comosus* (L.) Merr.) is one of the most important tropical fruits worldwide. It is primarily propagated clonally and exhibits high heterozygosity which can mask deleterious mutations, potentially leading to inbreeding depression and hindering breeding efforts. To address this, we conducted a comprehensive genome-wide analysis of 91 pineapple accessions and identified over 7.9 million high-quality SNPs. We utilized these SNPs to investigate the genetic structure and reproductive patterns of diverse cultivars and related varieties. Population structure analysis revealed a unique genetic makeup of *A. comosus* var. *microstachys* accessions and unidirectional gene flow from *A. comosus* var. *microstachys* into *A. comosus* var. *comosus*,* A. comosus* var. *bracteatus*, and *A. comosus* var. *erectifolius*. Among the cultivated pineapples, ‘Mordilona-related’ and ‘Smooth Cayenne’ cultivars showed unidirectional gene flow from ‘Singapore Spanish’, ‘Queen’, and the wild *A. comosus* var. *microstachys*. Heterozygosity patterns suggested predominantly asexual reproduction in ‘Queen’, ‘Singapore Spanish’, ‘Smooth Cayenne’, and *A. comosus* var. *microstachys* populations, while ‘Mordilona-related’ and *A. comosus* var. *bracteatus* populations might have experienced increased sexual reproduction or population expansion. We developed two SNP panels: one for germplasm identification and the other one for pedigree analysis. These resources will facilitate pineapple germplasm evaluation, diversity analysis, and informed breeding decisions for cultivar improvement.

**Supplementary Information:**

The online version contains supplementary material available at 10.1007/s00438-025-02275-1.

## Introduction

Pineapple (*Ananas comosus* (L.) Merr.) is one of the most important tropical fruits worldwide, with a world production of 29.36 million tons in 2022 (FAO 2023). It is cultivated predominantly for fresh consumption as well as processed products such as canned fruit and juice. Beyond its nutritional importance as a food source, pineapple also serves as a primary source of bromelain, a proteolytic enzyme with pharmaceutical and culinary applications, and its leaves and stems provide natural fiber. The USDA pineapple germplasm collection offers vital diverse genetic material for breeding programs. Comprehensive, genome-wide characterization of this collection is essential to strengthen conservation efforts and enhance its utilization in both research and breeding programs.

Originating in South America, pineapple was introduced to Europe during the 16th century by Portuguese and Spanish explorers, facilitating its global spread. Pineapple belongs to the genus *Ananas* of the family Bromeliaceae. The genus *Ananas* consists of two species: *A. comosus* (L.) Merrill and *A. macrodontes* Morren (Coppens d’Eeckenbrugge and Leal 2018). *A. comosus* encompasses five botanical varieties, of which *A. comosus* var. *comosus*, *A. comosus* var. *bracteatus*, and *A. comosus* var. *erectifolius* are domesticated, while *A. comosus* var. *microstachys* and *A. comosus* var. *parguazensis* remain in their wild state (Coppens d’Eeckenbrugge and Leal 2018). The edible pineapple belongs to the variety *A. comosus* var. *comosus*, while *A. comosus* var. *microstachys* is the most common wild pineapple widely distributed in neotropical regions, characterized by elongated leaves and small fruit. Morphological and genetic evidence suggests that *A. comosus* var. *comosus* was domesticated from *A. comosus* var. *microstachys*, with the Guianas and the Amazon likely serving as the epicenters of domestication (Duval et al. 2003). *A. comosus* var. *erectifolius* is morphologically very similar to *A. comosus* var. *microstachys*, except for the smooth character of the leaf. It is exclusively cultivated for fiber and has never been found in the wild. Intriguingly, DNA diversity studies have revealed unique connections between different clones of *A. comosus* var. *erectifolius* and *A. comosus* var. *microstachys*, suggesting parallel domestication events in various regions (Duval et al. 2001, 2003). *Ananas comosus* var. *bracteatus* is cultivated for fiber, fruit juice, and as a live hedge. Its geographic distribution overlaps with *A. macrodontes* in southeastern Brazil, exhibiting intermediate morphological and genetic characteristics between *A. comosus* and *A. macrodontes* (Baker and Collins 1939; Duval et al. 1997).

Cultivated pineapples have a relatively narrow genetic base, owing to their origins and propagation from a limited genetic reservoir. Pineapple cultivars are generally categorized into five distinct groups: Cayenne, Queen, Spanish, Mordilona, and Pernambuco. These groups are distinguished based on morphological characteristics, such as fruit size and shape, leaf color, peduncle length, number of slips, and presence of spines on leaf margins. ‘Smooth Cayenne,’ representative of the Cayenne group, once dominated global pineapple production in the 20th century, owing to its high yield and favorable characteristics for canning. The ‘Spanish’ group, although not widely cultivated globally, demonstrates adaptability to coastal peat environments and is primarily grown in South Asia countries. ‘Singapore Spanish’ remains significant in the processed fruit market and holds the second position as a canning cultivar, mostly grown in Malaysia. The ‘Queen’ variety, grown in tropical Indo-Pacific regions, South Africa, and Australia, is mainly for the fresh fruit market due to its unsuitability for canning. ‘Queen’ is known for its hardiness and disease resistance compared to ‘Smooth Cayenne’. ‘Mordilona’ and ‘Pernambuco’ hold regional importance, primarily grown in South American countries such as Brazil, Ecuador, Colombia, Venezuela, and Peru.

The pineapple breeding system involves both sexual reproduction and clonal selection. Vegetative propagation is the dominant reproduction form in nature owing to the hardiness and desiccation resistance of vegetative propagules, while sexual reproduction is restricted to breeding purposes (Duval et al. 1997). Recent genomic studies reveal the pivotal role of sexual selection during the early phases of pineapple domestication (Ming et al. 2015; Chen et al. 2019). While *A. comosu*s var. *comosus* exhibits robust self-incompatibility, other varieties possess a milder version of this trait, sometimes producing a few seeds from self-fertilization. The prevalence of vegetative reproduction may have relaxed selective pressure on fertility, potentially fostering allogamy and self-incompatibility. Human preference for seedless fruits further reinforced this phenomenon. Consequently, *A. comosus* var. *comosus* clones, particularly those of important cultivars, demonstrate reduced fertility and heightened self-incompatibility compared to their wild *A. comosus* counterparts and cultivars that are not primarily cultivated for fruit production (Coppens d’Eeckenbrugge et al. 1992).

The lack of recombination in clonally propagated crops hinders the elimination of deleterious mutations, potentially leading to an accumulation of unfavorable alleles (Ramu et al. 2017; Zhou et al. 2017; Chen et al. 2019). Furthermore, clonally propagated plants commonly exhibit a high level of heterozygosity (Ming et al. 2015; Ramu et al. 2017; Chen et al. 2019), which can mask deleterious mutations, allowing them to accumulate silently. These mutations, once homozygous during breeding, contribute to severe inbreeding depression. Inbreeding depression has been observed in ‘Smooth Cayenne’, one of the major parental lines used in pineapple breeding programs worldwide (Collins 1960). Addressing these challenges necessitates a thorough genome-wide evaluation of genetic variation present within germplasm collections. Such comprehensive assessments offer invaluable insights critical for informed decisions regarding the selection of optimal breeding materials.

Different statistical and analytical approaches have been used to assess clonality in populations, many of which are derived from biodiversity statistics such as the Shannon and Simpson indices and richness indices of genotypes (Arnaud-Haond et al. 2005, 2007). Among these approaches, the index of genotypic richness R has been widely used for over a decade (Arnaud-Haond et al. 2005). However, this index heavily relies on the sampling strategy and marker density (Arnaud‐Haond et al., 2007; Gorospe et al. 2015). With the advancement of genomic sequencing, genetic diversity indices have been widely used in ecological and evolutionary studies. Genetic diversity indices mathematically characterize the genetic compositions and variations and their deviations from Hardy–Weinberg equilibrium (HWE) (De Meeûs et al. 2007), as well as the assessment of linkage disequilibrium (Navascués et al. 2010). The *F*_*is*_ value reveals the excess of heterozygotes within a population and is considered as a strong indicator of the rate of clonal reproduction (Wei et al. 2012; Drott et al. 2020). *F*_*is*_ quantifies the deviation from HWE, and has proven valuable in evaluating the prevalence of asexual reproduction across diverse plant species (Gatto et al. 2013; Ho et al. 2019; Sharif et al. 2020; da Cunha et al. 2022; Huanel et al. 2022).

In this study, we analyzed the genome sequencing and resequencing data from 91 pineapple germplasm accessions to examine the genetic structure and reproductive mechanisms of diverse cultivars and wild varieties through population genomics approach. Additionally, we developed SNP panels for genetic characterization of pineapple germplasms. These resources will be valuable for researchers and breeders conducting genomics and molecular breeding experiments, such as assessments of genetic diversity, QTL mapping, and genomic selection.

## Materials and methods

### Variant calling of genome resequencing data

Genome sequencing and resequencing data of 91 pineapple germplasm accessions (Ming et al. 2015; Chen et al. 2019) were used for variant calling. These accessions encompassed 68 *Ananas comosus* var. *comosus* accessions, 8 *A. comosus* var. *bracteatus* accessions, 11 *A. comosus* var. *microstachys* accessions, 2 *A. comosus* var. *erectifolius* accessions, and 2 *Pitcairnia* spp. (*Pitcairnia gracilis* and *P. punicea*) accessions serving as outgroups. This dataset comprised approximately 5.0 billion 150-250 bp paired-end Illumina reads. Raw reads were filtered to remove low quality reads using Trimmomatic v0.39 (Bolger et al. 2014). Filtered reads were then aligned to the unmasked F153 pineapple reference genome using BWA-MEM aligner with default settings (Li and Durbin 2009). Further refinement included filtering out potential PCR duplicates, single-end mapped reads, and improperly paired reads. This filtering process was performed using samtools (Li et al. 2009) and picard (http://broadinstitute.github.io/picard*)* to ensure the accuracy of subsequent SNP calling.

The genetic variations were identified using the Genome Analysis Toolkit (GATK v.4.2.2.0) (McKenna et al., 2010) with an adapted best practice workflow. The processed BAM files were used to call raw variations for each accession independently using both HaplotypeCaller implemented in the GATK package (McKenna et al., 2010) and BCFtools program (Danecek et al. 2021) with default settings. Only variations concordantly called by both HaplotypeCaller and BCFtools were retained. Subsequently, SNPs and INDELs were separated and subjected to GATK-recommended hard filtering. SNPs were filtered using the parameters “ReadPosRankSum < -8.0|| QD < 2.0|| FS > 60.0|| SOR > 3.0|| MQ < 40.0|| MQRankSum < -12.5”, while INDELs were filtered using the parameters “ReadPosRankSum < -20.0|| QD < 2.0|| FS > 200.0|| SOR > 10.0”. This filtration yielded the final raw set of SNPs and INDELs for each accession.

Individual VCF files of the 91 accessions were merged using BCFtools (Danecek et al. 2021). To ensure the accuracy of variations and their frequencies, missing genotypes in the merged VCF file were recalibrated. To facilitate population genetics analysis, read depths at each position were tallied from the final BAM files using samtools to assist the calibration of missing genotypes in the merged SNP file.

### Heterozygosity and SNP annotations

We identified a total of 21,557,147 SNPs. Genome-wide coverage and heterozygosity were estimated for each accession, allowing us to calculate individual heterozygosity metrics. SNP annotation was performed using the SNPEff v.5.1 (Cingolani et al. 2012) with the pineapple F153 gene models (Ming et al. 2015). These SNP annotations were categorized based on their genomic positions and impact (nonsynonymous or synonymous).

The raw SNP dataset was further filtered for population analyses. SNPs with a minor allele frequency below 0.02, missing data exceeding 10%, or non-biallelic variation were removed. This yielded a final set of 7,944,346 high-quality SNPs, which were then subjected to comprehensive annotation analysis.

### Population structure analysis

We analyzed the genomic ancestry of 89 *Ananas* accessions using the curated set of high-quality SNPs. ADMIXTURE v1.3.0 (Alexander et al. 2009) was used to investigate genome-wide ancestry and admixture patterns. A range of ancestral components (K = 3 to 12) was systematically evaluated. Cross-validation (CV) error was used to identify the optimal K value, whereby the K value associated with the lowest CV error was deemed optimal. In our analysis, the optimal model identified six distinct ancestral genetic components in the pineapple population. To visually represent the population structure and admixture across the main pineapple varieties and cultivars, we generated figures illustrating ancestry components at K = 5, 6, and 7.

### Estimation of inbreeding coefficient (*F*_*is*_) and tajima’s D

Based on the population structure analysis, we identified representative ancestral components for each distinct *A. comosus* variety and the four domesticated pineapple cultivars (‘Singapore Spanish’, ‘Queen’, ‘Mordilona-related’, and ‘Smooth Cayenne’). Accessions displaying over 70% genetic congruence with their respective variety-representative components (at K = 5, 6, and 7) were classified as the definitive accessions for these varieties/cultivars. Accessions exhibiting admixed genetic profiles were categorized as hybrids.

Due to limited samples from *A. comosus* var. *erectifolius* and *Pitcairnia* spp., our analysis primarily focused on seven populations: *A. comosus* var. *bracteatus*, *A. comosus* var. *microstachys*, four domesticated pineapple cultivars (*A. comosus* var. *comosus*), and hybrid groups (‘Mordilona-related’ and ‘Smooth Cayenne’). To assess genetic diversity within these populations, we calculated inbreeding coefficient (*F*_*is*_) and Tajima’s D for each of these seven populations. *F*_*is*_ quantifies deviations from Hardy-Weinberg Equilibrium (HWE) using the following formula:

$$\:{F}_{is}=1-\frac{{f}_{Aa}}{2*{f}_{A}*{f}_{a}}$$

where *f*_*Aa*_ represents the proportion of observed heterozygosity, and *f*_*A*_ and *fa* represent the frequencies of the two alleles. Tajima’s D evaluates the excess or lack of rare alleles by comparing two estimators of genetic diversity: the average count of pairwise differences and the number of segregating sites. The *F*_*is*_ and Tajima’s D were calculated for each SNP locus. We then compared their distributions across the seven populations.

### SNP panel design for pineapple germplasm evaluation

From the initial raw SNP dataset, we extracted SNPs relevant to the 68 *A. comosus* var. *comosus* accessions and then applied stringent filtration. We eliminated SNPs residing on unanchored scaffolds or contigs. SNPs with any missing data were also excluded to ensure equal contribution of each SNP. Minor allele frequency (MAF) filtration was applied, with only SNPs having MAF ≥ 0.02 being retained.

To capture genetic diversity among pineapple cultivars and accessions, we formulated SNP panel A through the following steps: (1) removal of SNPs within repetitive sequences and their 100 bp flanking regions, (2) identification of unique intra-cultivar genotypes, yielding a SNP set for each of the four *A. comosus* var. *comosus* groups, (3) random selection of one SNP for SNPs sharing identical genotypic patterns across 68 accessions, (4) ranking SNPs by assessment of genotype differences between representative cultivars and other accessions, (5) selection of the top 50 SNPs showing the highest genotypic differences for each of the four *A. comosus* var. *comosus* groups, 6) removal of SNPs within distances ≤ 100 bp, and retaining a single SNP within100 kb. These SNPs were then merged to form SNP panel A. We subsequently conducted multidimensional scaling (MDS) analysis on SNP panel A using the “MASS” package within the R program.

In parallel, following MAF filtration, SNPs with any heterozygous genotypes across the 68 accessions were discarded. SNPs within distance ≤ 100 bp were removed, retaining only one SNP within 100 kb. Similarly, only one SNP was retained for SNPs sharing identical genotypic patterns across 68 accessions. This yielded SNP panel B, composed solely of homozygous SNPs, tailored for pedigree analysis.

## Results

### Genome-wide SNP identification in pineapple

In this study, we utilized genome sequencing and resequencing data from 91 accessions representing diverse pineapple varieties (Ming et al. 2015; Chen et al. 2019). This collection included 68 accessions of *A. comosus* var. *comosus*, 8 accessions of *A. comosus* var. *bracteatus*, 11 accessions of *A. comosus* var. *microstachys*, 2 accessions of *A. comosus* var. *erectifolius*, and 2 accessions of *Pitcairnia* (*P. gracilis* and *P. punicea*), which were included as outgroups (Supplementary Table 1). The cultivated pineapple accessions (*A. comosus* var. *comosus*) represent four major pineapple groups: ‘Smooth Cayenne’, ‘Queen’, ‘Singapore Spanish’, and ‘Mordilona-related’.

**Fig. 1:**
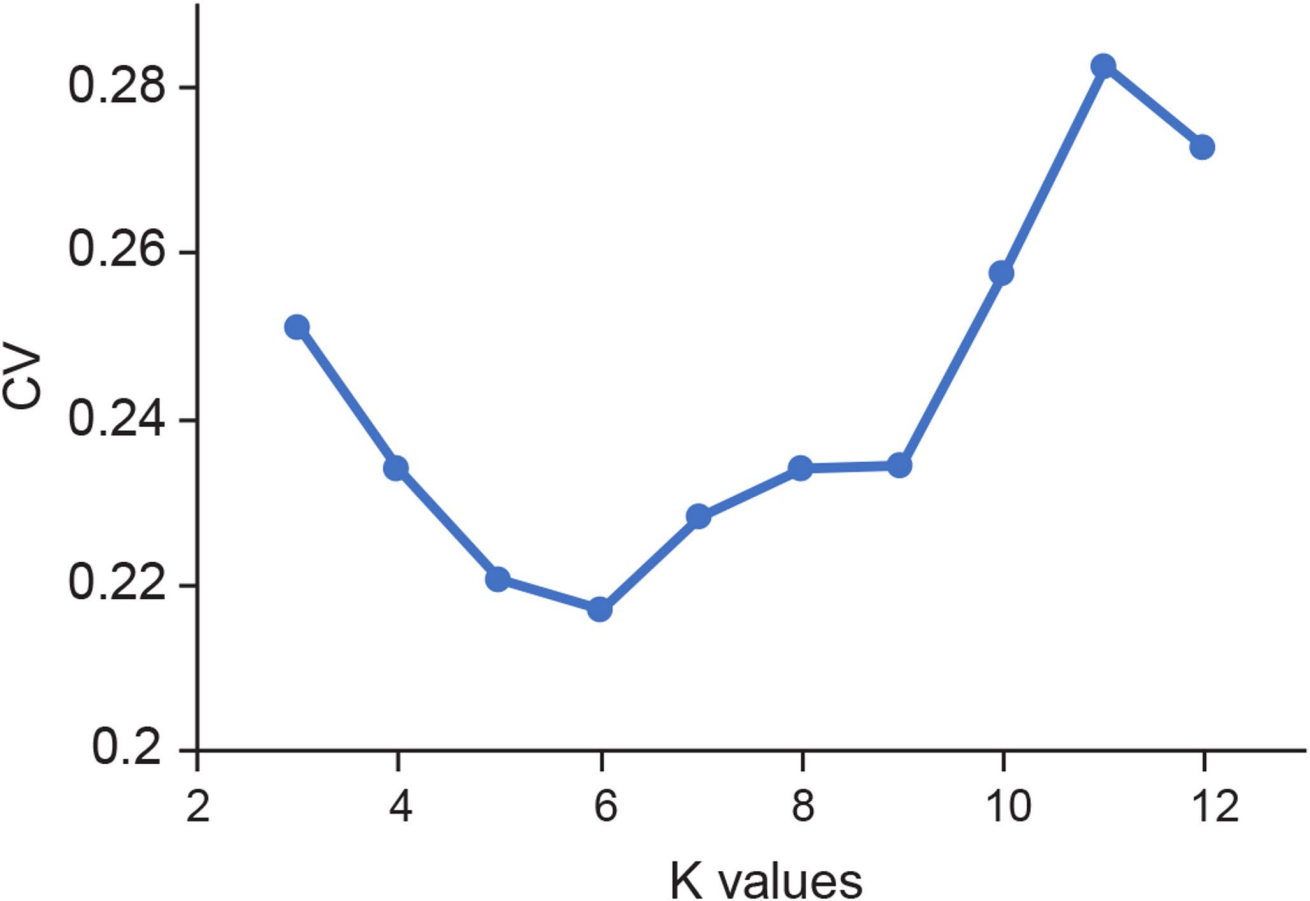
Overview of SNPs in pineapple. **a** Heterozygosity distribution of 91 pineapple accessions. The x-axis displays all 91 accessions, arranged in ascending order based on their individual heterozygosity levels. Detailed information can be found in Supplementary Table 1. **b** Annotation of SNPs. SNPs are categorized based on their genomic locations and predicted impact (nonsynonymous or synonymous changes). Only high-quality SNPs are presented here after removing minor alleles (allele frequency < 0.02) and SNPs with missing data > 10%

Initially, we identified a total of 22,557,147 SNPs across the 91 accessions before filtering based on allele frequency or missing data. The 68 cultivated pineapple accessions contributed 12,917,187 of these raw SNPs. Consistent with previous studies (Ming et al. 2015; Chen et al. 2019), our analysis revealed elevated heterozygosity levels in *Ananas* species, ranging from 0.20 to 1.46% across the accessions (Fig. 1a; Supplementary Table 1). The average heterozygosity of the 68 cultivated pineapple accessions was approximately 0.67% (Fig. 1a; Supplementary Table 1). *A. comosus* var. *bracteatus* exhibited the highest average heterozygosity (1.13%), while *A. comosus* var. *microstachys* displayed the lowest average heterozygosity (around 0.45%) (Fig. 1a; Supplementary Table 1).

After removing SNPs with a MAF < 0.02 and missing data ˃ 10%, we obtained a high-quality dataset of 7,944,346 SNPs. The majority (74.9%, 5,954,186 SNPs) were located outside of genic regions, including downstream (16.5%), upstream (25.0%), and intergenic regions (22.5%) (Fig. 1b; Supplementary Table 2). The genic SNPs were distributed in exons (8.2%), introns (14.4%), and untranslated regions (UTRs) (2.4%) (Fig. 1b; Supplementary Table 2). SNP annotation revealed that 3.6% (288,346) were synonymous variations, while a slightly higher proportion (3.8%, 302,033) were predicted to cause missense amino acid changes. The SNP dataset also included annotations for alterations in start codons (9,241), disruptions of stop codons (6,541), and splice site changes (44,085) (Fig. 1b; Supplementary Table 2).

### Population structure and hybridization patterns of pineapple

**Fig. 2:**
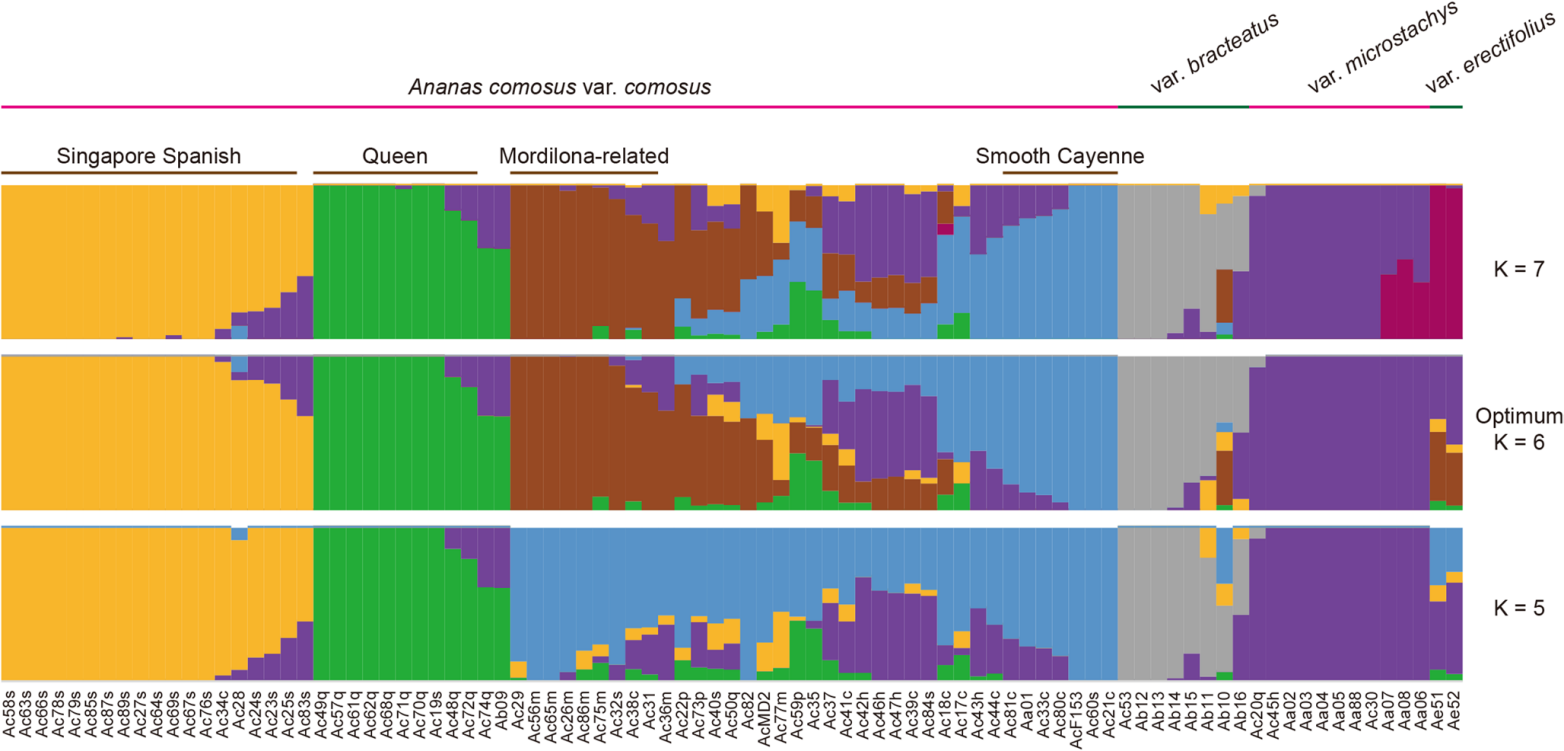
Cross validation (CV) error plot for ADMIXTURE analysis with *K* value ranging from 3 to 12

To understand gene flow in pineapples, we analyzed population structure across a range of varieties and cultivars. Using the ADMIXTURE program (Alexander et al. 2009), we inferred ancestral genetic components based on a dataset of approximately 7.9 million high-quality SNPs. This led to the selection of an optimal model comprising six distinct genetic components (Fig. 2). Despite significant heterozygosity among pineapple accessions, genome-wide inferences revealed a clear genetic structure.

**Fig. 3:**
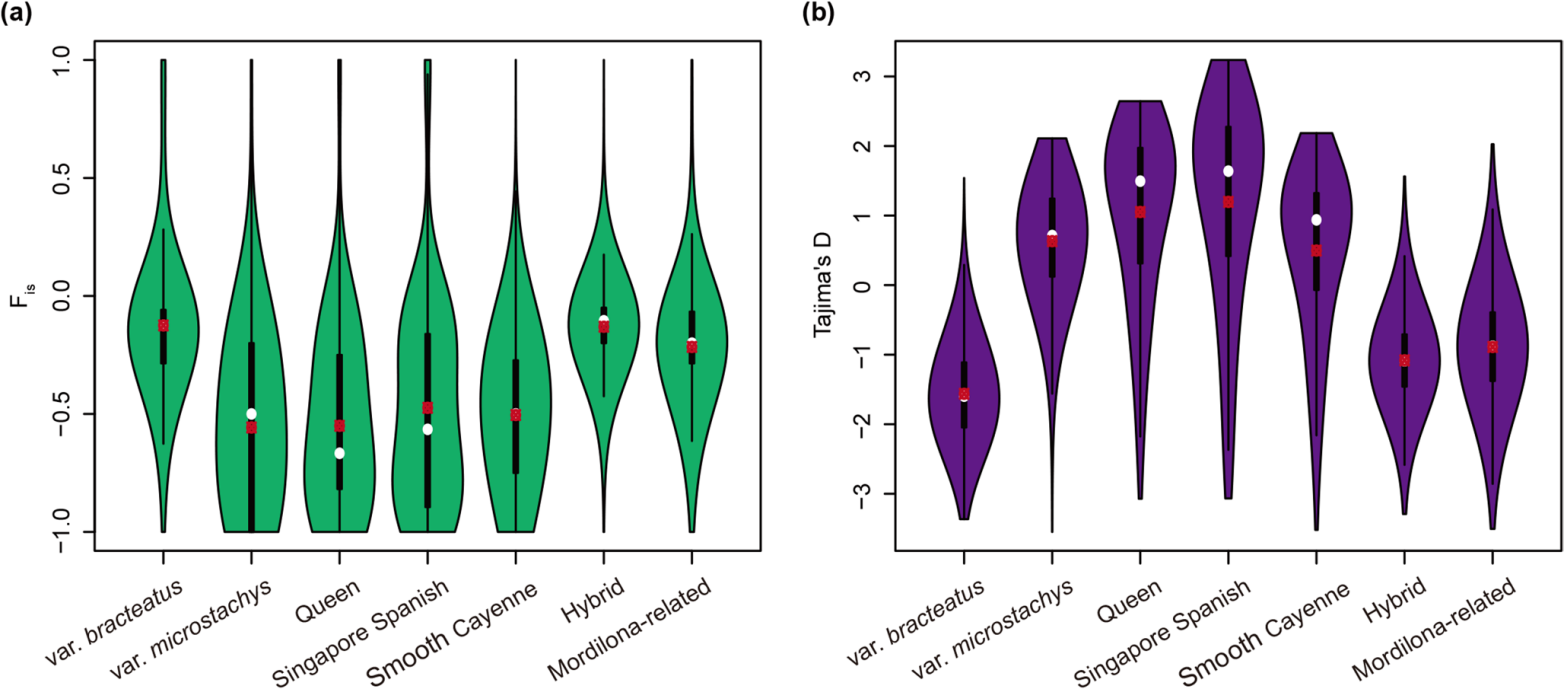
The population structure of 89 *Ananas comosus* accessions. The population structures generated with K = 5, 6, and 7 are presented. The cultivated pineapple accessions are grouped based on their genetic components. The accessions were assigned to representative groups only if they contain more than 70% of the characteristic genetic components of the representative population

*A. comosus* var. *microstachys* accessions displayed a unique genetic makeup, and unidirectional gene flow was observed from *A. comosus* var. *microstachys* into *A. comosus* var. *comosus*, *A. comosus* var. *bracteatus*, and *A. comosus* var. *erectifolius*. *A. comosus* var. *bracteatus* exhibited its own genetic components with some gene flow from *A. comosus* var. *comosus* (Fig. 3). Within *A. comosus* var. *comosus*, ‘Singapore Spanish’ and ‘Queen’ cultivars displayed largely uniform genetic composition within their respective populations. Minimal hybridization was evident in these cultivars, with only a few accessions showing gene flow from wild varieties, primarily *A. comosus* var. *microstachys* (Fig. 3). In contrast, ‘Mordilona-related’ and ‘Smooth Cayenne’ cultivars showed a significant proportion of accessions with mixed genetic components (Fig. 3), suggesting frequent hybridization. This observation is consistent with the population structure analysis at K = 5, which revealed shared principal ancestral components and a close genetic relationship between these two cultivars (Fig. 3). In addition, ‘Mordilona-related’ and ‘Smooth Cayenne’ cultivars showed primarily unidirectional gene flow from ‘Singapore Spanish’, ‘Queen’, and the wild *A. comosus* var. *microstachys* (Fig. 3).

Based on the population structures, we inferred potential gene flow patterns among pineapple varieties and cultivars. Additionally, we identified representative genetic components for each group. Accessions were classified as hybrids if they possessed less than 70% of the characteristic genetic components of their respective populations (Fig. 3). These findings provide a basis for exploring pineapple evolutionary history and developing tools for pineapple genetic research.

### Genetic effects of reproductive systems in pineapple

High heterozygosity is often observed in crop species that undergo outcrossing or clonal propagation. In this study, we examined heterozygosity in pineapple populations using key population genetics metrics: *F*_*is*_ and Tajima’s D. These metrics allow for effective differentiation between the influences of outcrossing, clonal propagation, and demographic history on the observed elevated heterozygosity.

**Fig. 4:**
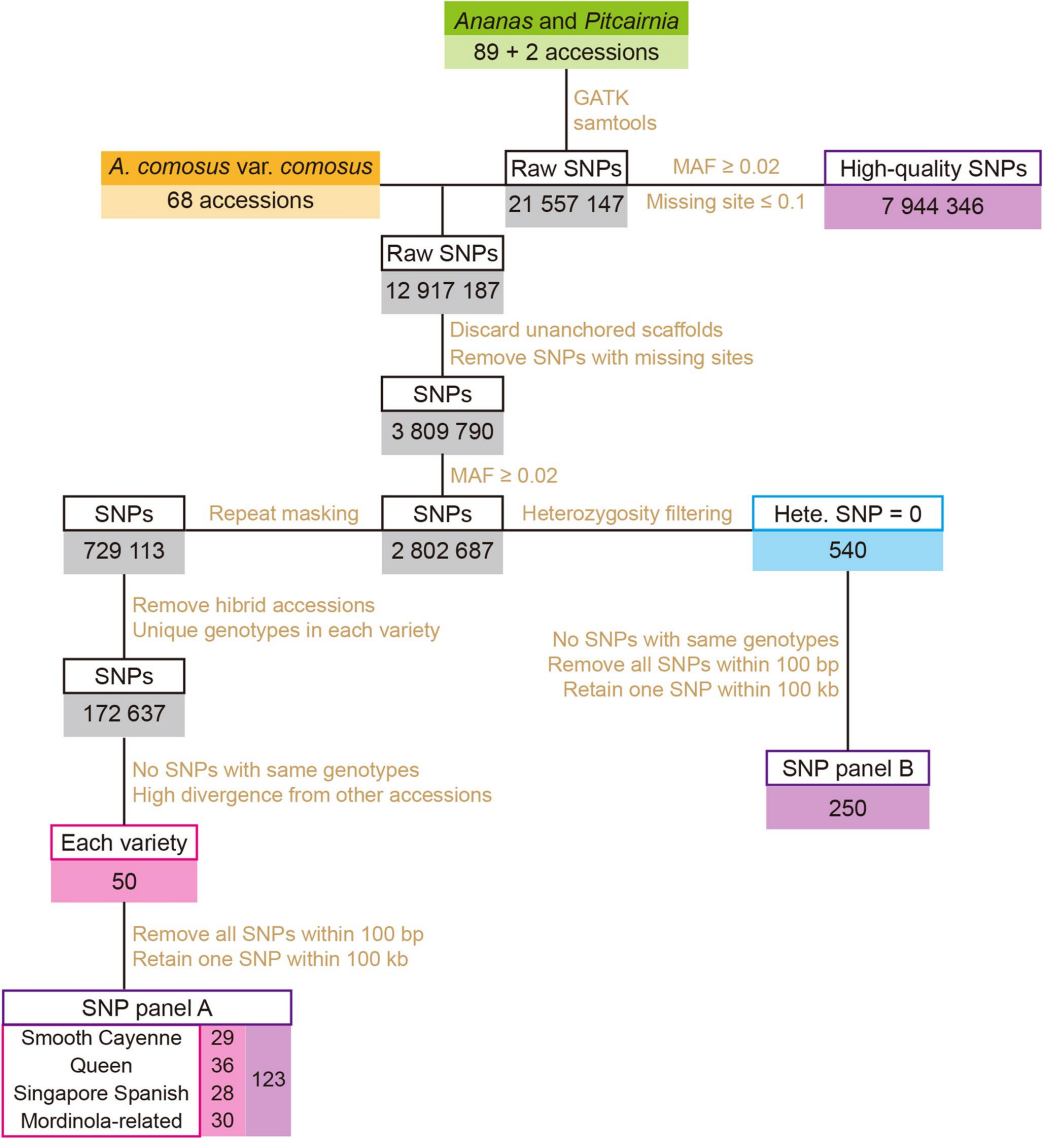
Estimation of inbreeding coefficient (*F*_*is*_) and Tajima’s D in pineapple populations. **a** Estimation of inbreeding coefficient *F*_*is*_ in pineapple populations. The violine plots present the distribution of *F*_*is*_ values in pineapple populations. Red dots denote the mean values of *F*_*is*_. *b* Estimation of Tajima’s D in pineapple populations

*F*_*is*_ estimates deviations from the HWE, while Tajima’s D assesses the excess or lack of rare alleles. *F*_*is*_ and Tajima’s D were estimated across seven populations, each with at least five accessions, including four *A. comosus* var. *comosus* cultivar groups (‘Singapore Spanish’, ‘Queen’, ‘Mordilona-related’, and ‘Smooth Cayenne’), a hybrid group between ‘Smooth Cayenne’ and ‘Mordilona-related’, and two other *A. comosus* varieties: *A. comosus* var. *bracteatus* and *A. comosus* var. *microstachys* (Figs. 3 and 4). Among these, ‘Queen’, ‘Singapore Spanish’, ‘Smooth Cayenne’, and *A. comosus* var. *microstachys* demonstrated much lower *F*_*is*_ values, indicative of an exceptional excess of heterozygous genotypes (Fig. 4a). The negative *F*_*is*_ values suggest these populations predominantly underwent clonal propagation with limited outcrossing. In contrast, the hybrid population closely aligned with HWE, with its two progenitors, ‘Smooth Cayenne’ and ‘Mordilona-related’, exhibited divergent *F*_*is*_ patterns—greater excess of heterozygosity in ‘Smooth Cayenne’ and less heterozygosity in ‘Mordilona-related’ (Fig. 4a). *F*_*is*_ estimation also revealed distinct patterns between *A. comosus* var. *bracteatus* and *A. comosus* var. *microstachys*, suggesting different evolutionary trajectories in accumulating genetic diversity. *F*_*is*_ values closer to zero in ‘Mordilona-related’, the hybrid, and *A. comosus* var. *bracteatus* populations suggest relatively less clonal propagation than the other four populations.

Tajima’s D estimates, which reflect rare allele frequency and demographic history, supported the *F*_*is*_ findings (Fig. 4b). Positive Tajima’s D values in the ‘Queen’, ‘Singapore Spanish’, ‘Smooth Cayenne’, and *A. comosus* var. *microstachys* populations indicate an excess of common alleles and a scarcity of rare alleles. This pattern may result from reduced outcrossing and increased asexual reproduction, leading to elevated heterozygosity and fewer rare alleles. Conversely, negative Tajima’s D values were observed in the ‘Mordilona-related’, hybrid, and *A. comosus* var. *bracteatus* populations, suggesting an excess of rare alleles. This result is consistent with their lower *F*_*is*_ deviations (Fig. 4a), implying these populations likely underwent sexual reproduction or population expansion, both of which increase rare allele frequency and reduce deviations from HWE.

### SNP panel design for pineapple germplasm evaluation

To serve future needs in pineapple germplasm evaluation and utilization, we developed two small SNP panels that can be used for genotype identification and pedigree analysis. Reducing the number of SNPs enhances computational efficiency and storage requirements while preserving the power to discriminate between individuals.

**Fig. 5:**
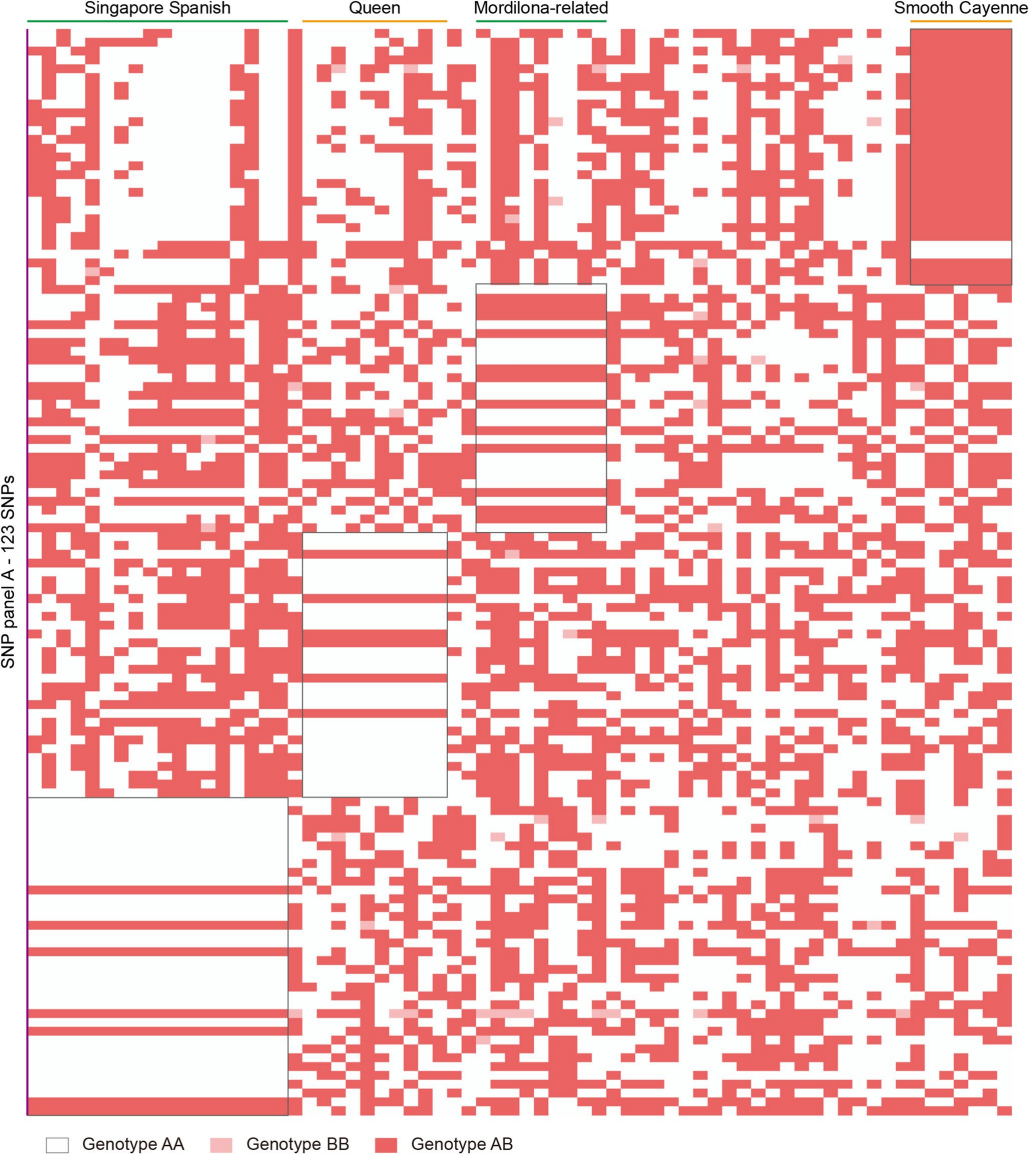
Development workflow of the pineapple SNP panels

**Fig. 6:**
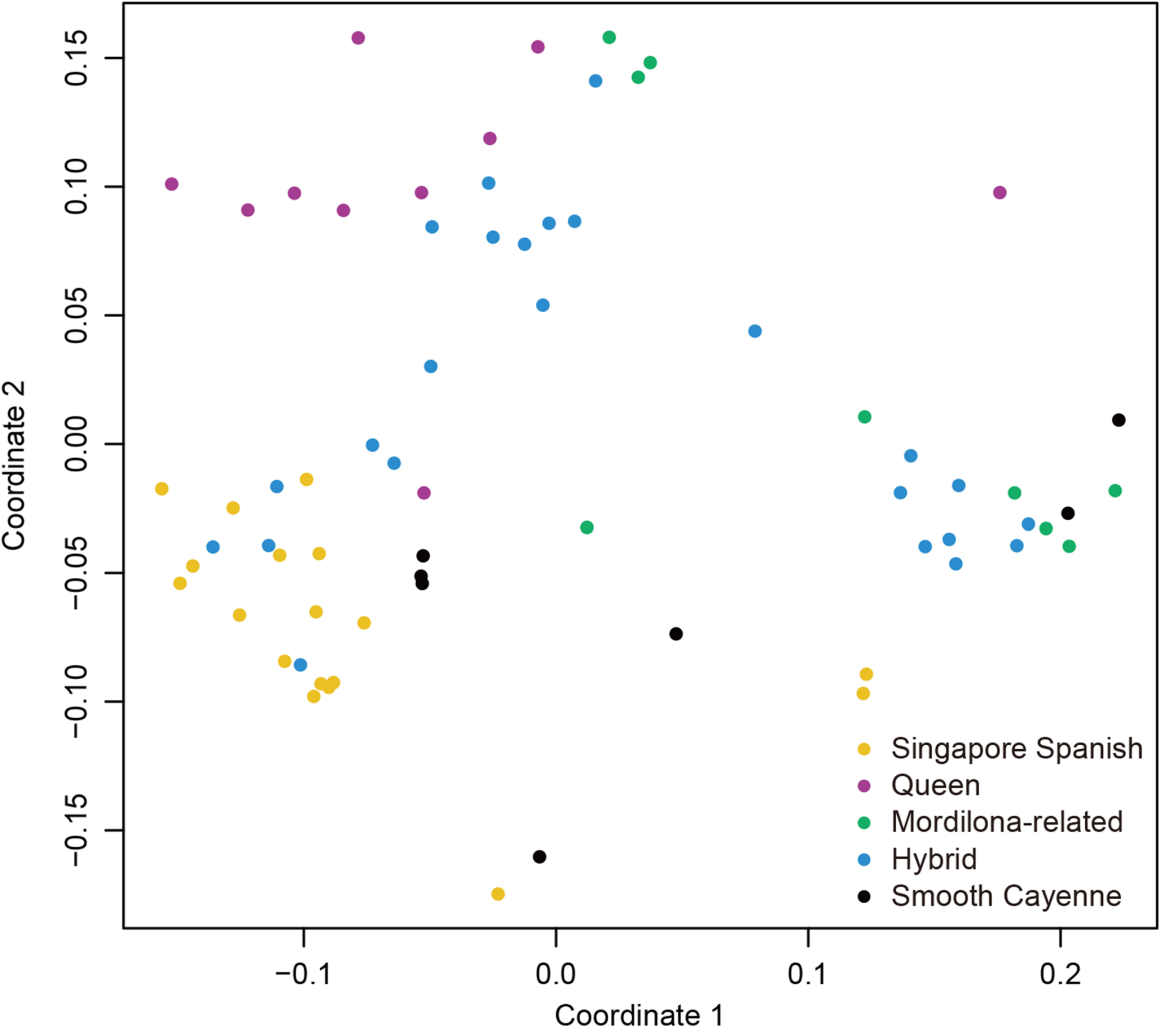
Heatmap of genotyping patterns of SNP panel A across 64 cultivated pineapple accessions. White, pink, and red colors indicate AA, BB, and AB genotypes, respectively. Detailed information is available in Supplementary Table 4

**Fig. 7:**
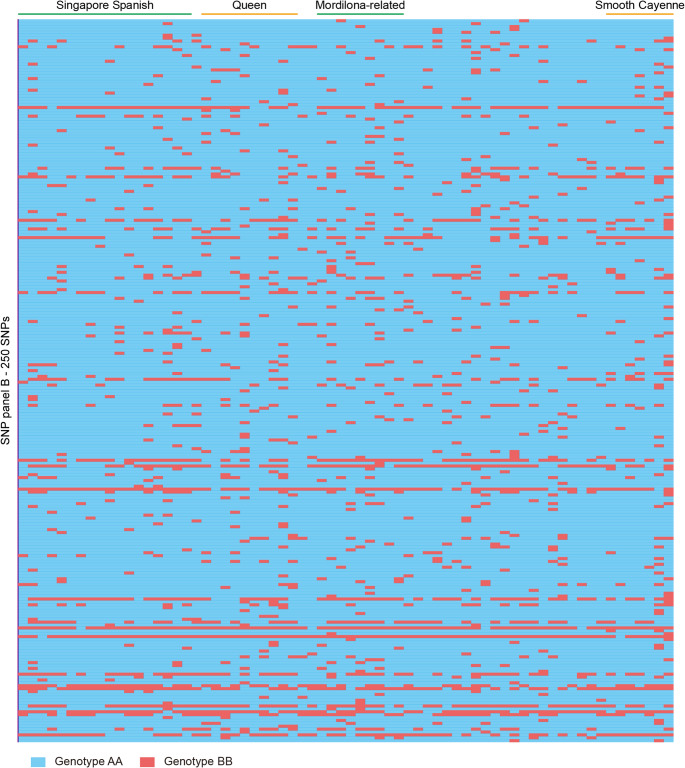
Multidimensional scale (MDS) clusters of the genotyping data of 64 cultivated pineapple accessions using SNP panel A

We extracted 12.9 million raw SNPs from the 68 cultivated pineapple accessions (Fig. 5). After mapping and filtering based on MAF ≥ 0.02, we retained 2.8 million high-quality SNPs (Fig. 5). Further refinement of this SNP pool resulted in two specialized SNP panels designed for genotype identification and pedigree analysis (Fig. 5). SNP Panel A is designed for distinguishing cultivars and accessions. To avoid primer design issues related to repetitive sequences, we masked SNPs located within repetitive sequences and their 100-base-pair flanking regions, leaving approximately 927,000 SNPs (Fig. 5). To identify cultivar-specific SNPs, we generated four separate SNP sets, one for each of the four major pineapple groups: ‘Smooth Cayenne’, ‘Queen’, ‘Singapore Spanish’, and ‘Mordilona-related’ (Figs. 3, 5 and 6). After stringent filtering, the top 50 cultivar-specific SNPs exhibiting the greatest genotypic divergence from other cultivar groups were selected for each representative cultivar group (Figs. 5 and 6). Final filtering steps included removing SNPs within distances less than 100 bp and retaining a single SNP per 100-kb region, resulting in a 123-SNP panel distributed across four major cultivar group (Figs. 5 and 6; Supplementary Table 3). The final SNP Panel A comprised of 29 SNPs for ‘Smooth Cayenne’, 36 for ‘Queen’, 28 for ‘Singapore Spanish’, and 30 for ‘Mordilona-related’ cultivars (Figs. 5 and 6; Supplementary Table 3). Among these, 75 SNPs are in genic regions, encompassing 25 intronic variations, 16 synonymous mutations, and 18 nonsynonymous mutations (Supplementary Table 4). MDS visualization confirmed that the SNP Panel A can effectively distinguish cultivars and cluster these cultivar groups (Fig. 7).

**Fig. 8:**
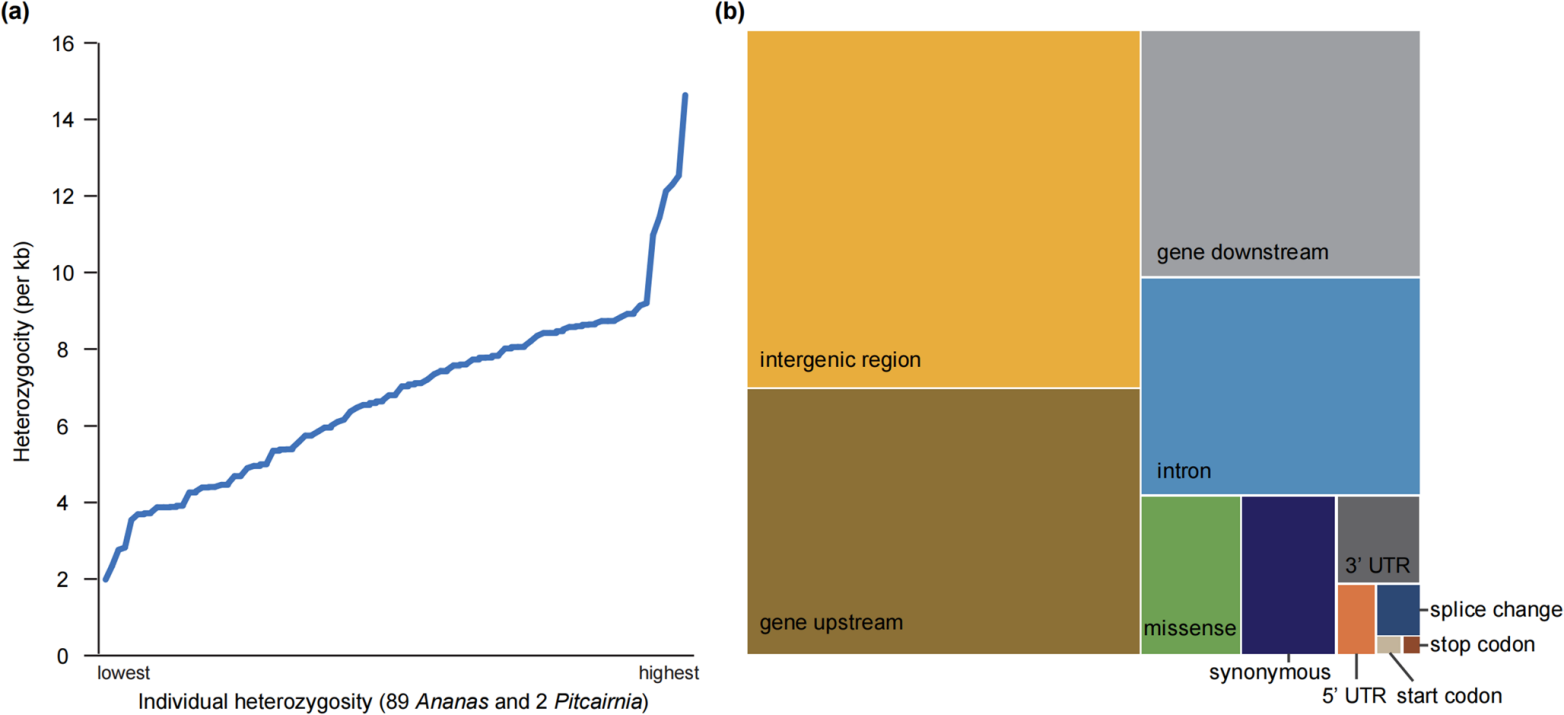
Heatmap of genotyping patterns of SNP panel B across 64 cultivated pineapple accessions. Each color represents a specific genotype: blue for AA and red for BB. Detailed genotyping data is available in Supplementary Table 6

We applied filtering criteria (MAF ≥ 0.02, heterozygosity frequency = 0) to the initial 2.8 million high-quality SNPs and obtained 540 SNPs (Fig. 5). Further filtering steps, including removal of SNPs exhibiting identical genotypes and SNPs with distances less than 100 bp, and retention of a single SNP per 100 kb region, were applied and resulted in 250 SNPs (Figs. 5 and 8; Supplementary Table 5). These 250 SNPs formed SNP Panel B. Among the 250 SNPs, 53 are located within genic regions, 121 reside within transposons, and 76 are positioned in intergenic regions (Fig. 8; Supplementary Table 6).

These two SNP panels developed in this study fulfill distinct roles: SNP Panel A facilitates the identification of cultivars and accessions, whereas SNP Panel B can assist to trace parental lineage. These two SNP panels also offer valuable resources for various applications in pineapple research and improvement, including germplasm assessment, genetic diversity analysis, population evaluation, and genome-informed breeding strategies.

## Discussion

Genomic evaluation of germplasm collections is essential for efficiently utilizing and preserving genetic diversity, accelerating crop improvement, and facilitating trait discovery and functional genomics studies. In this study, we leveraged population genomic data to gain deeper insights into the genetic variations and evolutionary histories that have shaped pineapple diversity.

Early studies on *Ananas* diversity resolved sub-species level classification but failed to adequately survey the genetic diversity, evolutionary history, and relationships of cultivated accessions. The low resolution of these polymorphisms also prohibited identifying genomic signatures of sexual and asexual selection. By analyzing genome sequencing and resequencing data from 91 *Ananas* accessions, we identified over 7.9 million high-quality SNP markers. These markers provide valuable resources for high-resolution assessments of genetic diversity, phylogeny, and population structure in *Ananas*. Utilizing these SNP markers, we investigated the population structure of *A. comosus*. *A. comosus* consists of five botanical varieties: *A. comosus* var. *comosus*, var. *bracteatus*, var. *erectifolius*, var. *microstachys*, and var. *parguazensis* (Coppens d’Eeckenbrugge and Leal 2018). Among these, *A. comosus* var. *microstachys* exhibits the highest degree of genetic diversity, with its natural distribution encompassing the entire species. Genetic and morphological studies suggested the central role of *A. comosus* var. *microstachys* as the wild ancestor of the domesticated *A. comosus* var. *comosus*, var. *bracteatus*, and var. *erectifolius* (Coppens d’Eeckenbrugge et al. 2018). Consistent to this hypothesis, our population structure analysis showed unidirectional gene flow from *A. comosus* var. *microstachys* into *A. comosus* var. *comosus*, *A. comosus* var. *bracteatus*, and *A. comosus* var. *erectifolius*. In addition, population structure analysis revealed a high degree of admixture between cultivars and wild accessions, suggesting extensive breeding and selection during domestication process. This is particularly prevalent in ‘Mordilona-related’, ‘Smooth Cayenne’, and the hybrid groups. Pineapple breeding programs primarily use pre-Columbian varieties, such as ‘Smooth Cayenne’, ‘Singapore Spanish’, ‘Queen’, as foundational parents, with ‘Smooth Cayenne’ predominantly used as a female parent. Our study revealed unidirectional gene flow from ‘Singapore Spanish’, ‘Queen’, and the wild *A. comosus* var. *microstachys* into ‘Mordilona-related’ and ‘Smooth Cayenne’, likely reflecting this established breeding strategy.

Genetic diversity within populations is largely structured by reproduction. *A. comosus* var. *comosus* exhibits strong self-incompatibility, while other *A. comosus* varieties possess weaker self-incompatibility (Brewbaker and Gorrez 1967; Coppens d’Eeckenbrugge et al., 1992). Some of the *A. comosus* var. *bracteatus* clones show full self-compatibility (Brewbaker and Gorrez 1967). Our analysis revealed that *A. comosus* var. *bracteatus* exhibited the least deviations from HWE and a negative Tajima’s D, likely due to its *self-compatibility*. In contrast, major *A. comosus* var. *comosus* populations showed the largest deviations from HWE and a positive Tajima’s D, which could be partially explained by their strong self-incompatibility.

We used the population genomics approach to understand the interplay of sexual and asexual reproduction during the domestication process. By analyzing *F*_*is*_ and Tajima’s D statistics, we found distinct patterns of genomic variation across the different cultivars and varieties. ‘Queen’, ‘Singapore Spanish’, ‘Smooth Cayenne’, and the wild *A. comosus* var. *microstachys* populations exhibit signatures of significant asexual reproduction (clonal propagation), leading to an increase in heterozygosity and a depletion of rare alleles. In contrast, the ‘Mordilona-related’, hybrid, and *A. comosus* var. *bracteatus* populations show a better fit to HWE and possess an excess of rare alleles, suggesting these populations might have undergone population expansion or possess adaptive mechanisms to reduce genetic load.

High heterozygosity in pineapple, arising from limited genomic recombination and inefficient purging of deleterious variations, has been widely documented (Ming et al. 2015; Chen et al. 2019; Zhu et al. 2022). High heterozygosity can mask deleterious mutations, potentially leading to accumulation of deleterious variations and subsequently causing inbreeding depression and hindering breeding efforts (Bosse et al. 2019; Dwivedi et al. 2023). While the four populations with predominantly asexual reproduction exhibit high heterozygosity, the ‘Mordilona-related’, hybrid, and *A. comosus* var. *bracteatus* populations appear to have mechanisms to mitigate the genetic load. We hypothesize that population expansion through sexual reproduction, evidenced by the excess of rare alleles, contributes to reducing heterozygosity and, consequently, the genetic load in these populations. The hybrid population, with its diverse ancestral components, likely originated from hybridization events and shows an intermediate pattern between the ‘Mordilona-related’ and ‘Smooth Cayenne’ populations.

Despite extensive genomic research on pineapple, there remains a need for informative molecular markers to facilitate rapid germplasm identification and breeding applications. Our study addressed this gap by developing two SNP panels. SNP panel A, comprising 123 SNPs, enables the complete differentiation of pineapple cultivars and accessions while preserving the population structure among the four major cultivars. SNP panel B, containing 250 homozygous segregation sites across 68 pineapple accessions, is specifically designed for pedigree analysis. These SNP panels represent valuable tools for pineapple germplasm management, accelerating breeding efforts, and ensuring traceability in the pineapple trade.

## Conclusions

Our analysis of 91 *Ananas* accessions yielded over 7.9 million high-quality SNP markers. These markers provide valuable resources for high-resolution assessments of genetic diversity, phylogeny, and population structure in *Ananas*. We found unidirectional gene flow from *A. comosus* var. *microstachys* into domesticated varieties, supporting its central role as the wild ancestor. Heterozygosity patterns suggested predominantly asexual reproduction in ‘Queen’, ‘Singapore Spanish’, ‘Smooth Cayenne’, and *A. comosus* var. *microstachys* populations, while ‘Mordilona-related’ and *A. comosus* var. *bracteatus* may have experienced more sexual reproduction or population expansion. We also developed two SNP panels for germplasm identification and pedigree analysis. These findings deepen our understanding of pineapple genetic diversity and offer crucial resources for future research and crop improvement.

## Electronic supplementary material

Below is the link to the electronic supplementary material.

Supplementary Material 1
